# Stimulus Driven Functional Transformations in the Early Olfactory System

**DOI:** 10.3389/fncel.2021.684742

**Published:** 2021-08-03

**Authors:** Carlotta Martelli, Douglas Anthony Storace

**Affiliations:** ^1^Institute of Developmental Biology and Neurobiology, University of Mainz, Mainz, Germany; ^2^Department of Biological Science, Florida State University, Tallahassee, FL, United States; ^3^Program in Neuroscience, Florida State University, Tallahassee, FL, United States

**Keywords:** adaptation, olfactory bulb, olfaction, olfactory receptor neurons, mitral and tufted cells

## Abstract

Olfactory stimuli are encountered across a wide range of odor concentrations in natural environments. Defining the neural computations that support concentration invariant odor perception, odor discrimination, and odor-background segmentation across a wide range of stimulus intensities remains an open question in the field. In principle, adaptation could allow the olfactory system to adjust sensory representations to the current stimulus conditions, a well-known process in other sensory systems. However, surprisingly little is known about how adaptation changes olfactory representations and affects perception. Here we review the current understanding of how adaptation impacts processing in the first two stages of the vertebrate olfactory system, olfactory receptor neurons (ORNs), and mitral/tufted cells.

## Introduction

Adaptation modulates the input-output transformation of a neuron or brain region based upon the recent history of an organism’s sensory experience (Barlow, 1961; Wark et al., 2007; Whitmire and Stanley, 2016; Weber and Fairhall, 2019; Benda, 2021). Nearly all sensory systems exhibit some form of adaptation in which prolonged exposure to a stimulus evokes a change in the neural response. Defining the functional transformation(s) that take place as a result of adaptation remains a fundamental field of study in neuroscience and a critical step in understanding sensory processing.

**Figure 1:**
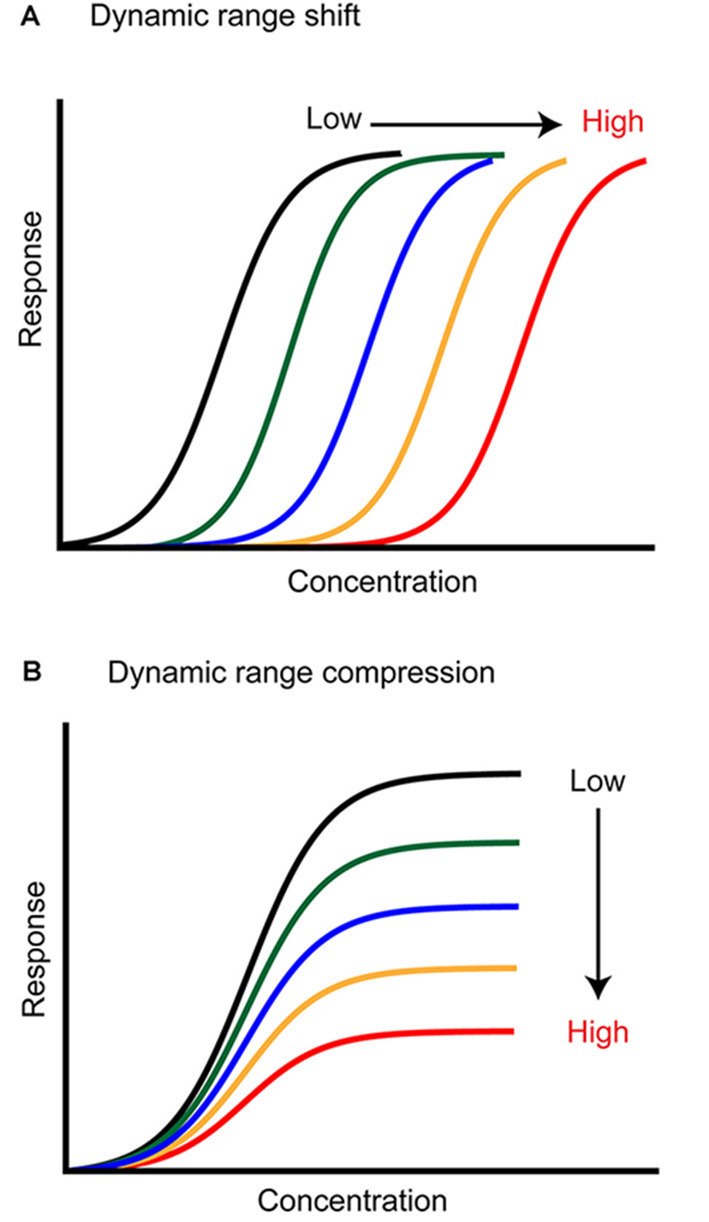
Two ways adaptation can transform neural responses. **(A)** Background stimuli of higher intensity (*color coded*) shift the neuron dynamic range toward higher concentrations, lowering its sensitivity to the stimulus. **(B)** Background stimuli of higher intensity (*color coded*) compress the dynamic range of the neuron. The neuron sensitivity is not changed, but the response saturates earlier, and the coding capacity is lower.

Sensory stimuli are experienced across a large range of intensities, but peripheral sensory neurons usually have a relatively small dynamic range. One function of adaptation is to shift this dynamic range toward relevant stimuli, a process that both expands the coding capacity of the sensory system and optimizes it (Barlow, 1961; Brenner et al., 2000; Wark et al., 2007; Whitmire and Stanley, 2016; Weber and Fairhall, 2019). For example, light can be experienced across ~10 log units of stimulus intensity (Skalicky, 2016), yet individual photoreceptors saturate across ~1–2 log units of light intensity located within their receptive field (Boynton and Whitten, 1970; Normann and Perlman, 1979; Valeton and van Norren, 1983; Perlman and Normann, 1998). Changing the background luminance level causes the sensitivity range of photoreceptors to shift along the intensity axis (Figure 1A; Boynton and Whitten, 1970; Normann and Perlman, 1979; Valeton and van Norren, 1983; Fain et al., 2001). This transformation allows a match between the photoreceptor coding capacity and the mean stimulus intensity. However, a changing neural response does not always reflect a shift in the dynamic range of a neuron. For example, the auditory system deals with a similar dynamic range problem (Viemeister, 1988; Chepesiuk, 2005) as auditory nerve fibers saturate within ~3–4 log units of sound intensity at their characteristic frequency, but the presence of a background stimulus causes reductions in auditory nerve spiking rather than a change in sensitivity (Figure 1B; Smith, 1977, 1979; Smith et al., 1983; but see Wen et al., 2009, 2012). Auditory neurons that exhibit dynamic range shifts exist, but at later stages of processing in the inferior colliculus and cortex (Dean et al., 2005; Nagel and Doupe, 2006; Watkins and Barbour, 2008). In general, matching the sensor’s dynamic range to the stimulus and optimizing its encoding in neural activity is the major goal of an adaptive change in sensory processing. But can we make similar observations in olfaction? Does adaptation adjust the dynamic range of olfactory neurons? And if so, which features of an odor stimulus are optimally encoded?

For many animals, the sense of smell is critical to recognize and locate food, mates, and dangers. Recognition requires the identification of a specific smell embedded in a complex chemical context. Localization, instead, requires the animal to extract information from a concentration profile that varies in time and space depending on the fluid-dynamic regime of the medium that transports these volatile molecules (Celani et al., 2014; Connor et al., 2018). The olfactory system, therefore, faces three challenges: (1) it has to segment the perception of a specific cue from other chemical stimuli present in the environment; (2) it has to maintain an invariant representation of the odor of interest when its concentration changes, and (3) at the same time, it has to recognize the direction of the changing gradient (Linster et al., 2007; Uchida and Mainen, 2007; Homma et al., 2009; Gottfried, 2010; Rokni et al., 2014). In this review, we summarize the functional changes in odor encoding driven by adaptation to sustained stimuli and discuss which cellular and network mechanisms could support **background segmentation**, **concentration invariance**, and **contrast coding** in the vertebrate olfactory system.

**Figure 2:**
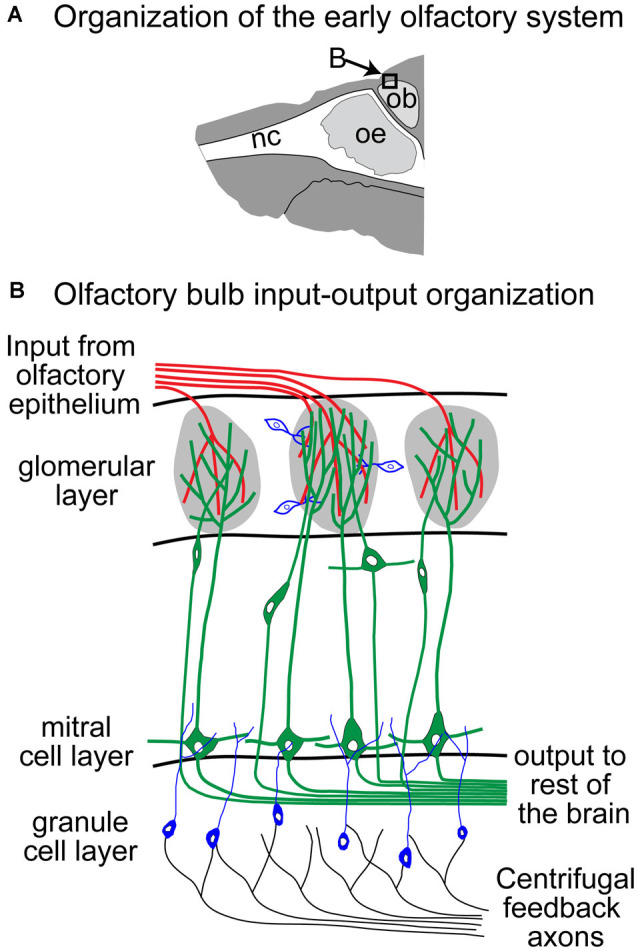
Schematic of the Olfactory Bulb (OB; adapted from Storace and Cohen, 2017). Panel **(B)** reflects an expansion of the box in panel **(A)**. nc, nasal cavity; oe, olfactory epithelium; ob, olfactory bulb.

Olfactory receptor neurons (ORNs) located in the olfactory epithelium send their axons to one or two glomeruli in the Olfactory Bulb (OB; Ressler et al., 1994; Vassar et al., 1994; Zapiec and Mombaerts, 2015) where they synapse on Mitral and Tufted Cells (MTCs), which then project to the olfactory cortices (Figure 2, *green processes*, Sosulski et al., 2011; Imamura et al., 2020). The OB input-output transformation is shaped by a complex synaptic network that includes many different populations of interneurons that surround each glomerulus (Parrish-Aungst et al., 2007; Nagayama et al., 2014; Burton, 2017), and granule cells that form dendro-dendritic synapses with the lateral processes of MTCs (Rall et al., 1966; Yokoi et al., 1995; Figure 2, *blue and black cells and processes*). Adaptation of ORNs has been extensively studied, although the functional role of the identified mechanisms remains a source of debate. Since odor stimuli are encoded combinatorially in populations of ORNs, the adaptation of olfactory representation likely involves coordinated changes across this population of cells, mediated by lateral connections within the OB. Moreover, in breathing animals, respiration plays a key role in modulating the sampling of the stimulus by controlling the times and durations of the odorous plumes that reach the sensory neurons. Here, we will compare results from *in vitro* experimental approaches that recorded odor responses from ORN somata in the epithelium, and *in vivo* experiments in breathing animals that quantified the response of the ORNs at their presynaptic site in the OB and the response of their postsynaptic MTCs. We will describe the concentration dependency of the response dynamics of these neurons and then focus on the changes in activity induced by repeated and sustained odor stimuli. These changes can occur over a wide range of timescales (seconds to days; Wang et al., 1993; Dalton and Wysocki, 1996; Chaudhury et al., 2010; Kass et al., 2016), but here we will focus on relatively short–term adaptation that is most likely to be relevant for odor navigation. Moreover, we focus on the vertebrate literature as adaptation in invertebrates has been recently covered (Brandão et al., 2021).

## Controversy and Consensus on ORN Adaptation

### Transient Firing Dynamics vs. Tonic Presynaptic Activity of ORN Odor Responses

**Figure 3:**
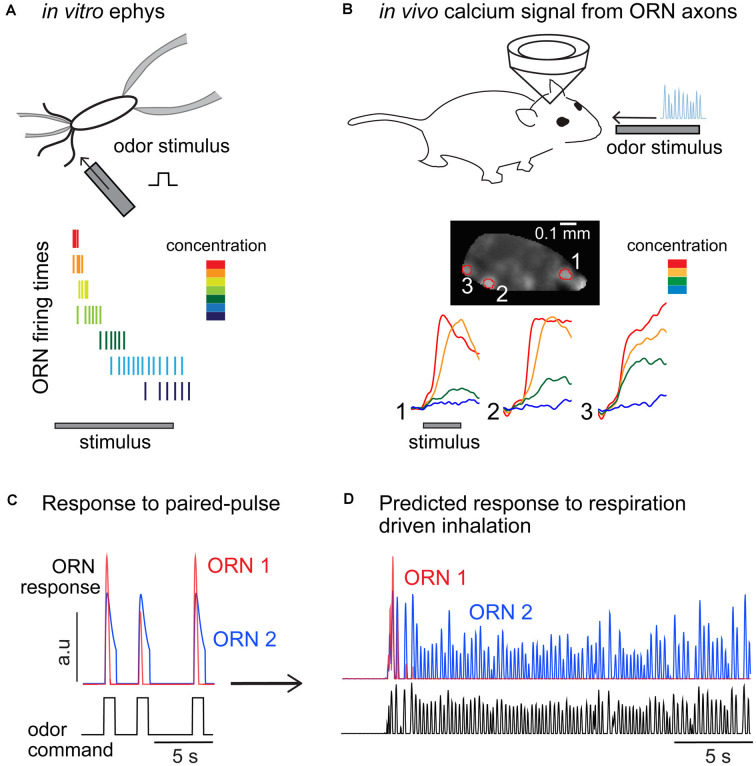
Comparisons of olfactory receptor neurons (ORNs) odor responses measured from the soma and at their glomerular projections in the olfactory bulb. **(A)** Odor stimuli elicit transient response dynamics as concentration increases, resulting in higher firing rates elicited in a very short time window and an overall decrease in the number of spikes fired. Cartoon inspired to data from Reisert and Matthews (1999). **(B)** Calcium measurements from ORN glomerular projections do not show transient dynamics nor reduced calcium levels at higher odor concentrations. Data reproduced from Storace et al. (2019). **(C)** Paired-pulse experiments showing two ORNs with different sensitivity and response dynamics. Consistent with published data, ORN1 is more sensitive to the odor and its response to a consecutive odor pulse is attenuated (2 s interstimulus). ORN1 response fully recovers in 4 s. ORN2 is less sensitive and does not show paired-pulse response attenuation. **(D)** Prediction of the response of the two ORNs to a continuous stimulus inhaled through several respiratory cycles (respiratory traces are unpublished data from D.A. Storace).

In vertebrates, olfactory stimuli are detected by ORNs that express one out of a large family (>1,000) of odorant receptors (ORs; Buck and Axel, 1991; Ngai et al., 1993; Ressler et al., 1994). ORNs extend cilia into the olfactory mucosa, where they are exposed to odor molecules. *In vitro* voltage or current clamp experiments from isolated ORNs allowed the identification of key players in the signaling cascade activated by an odor response (Schild and Restrepo, 1998). In short, the binding of an odor molecule to the receptor triggers the activation of a metabotropic pathway that leads to the increase of cAMP and opening of cyclic nucleotide gated (CNG) channels (Kleene, 2008). Calcium influx through CNG channels further enhances ORN depolarization by activating a Cl^−^ current (Reisert and Zhao, 2011). This amplification step is responsible for a large percentage of the odorant-induced transduction current. Consistent with classical models of receptor-ligand binding kinetics (Dougherty et al., 2005), the amplitude of the transduction current associated with this signaling cascade exhibits a sigmoidal monotonic relationship with odor concentrations, with most studies reporting a dynamic range of ~10-fold (Firestein et al., 1993; Menini et al., 1995; Kurahashi and Menini, 1997; Ma et al., 1999; Reisert and Matthews, 2001b; but see Grosmaitre et al., 2006). Before additional mechanisms kick in, ORN peak firing rates also show a similarly narrow dynamic range (Reisert and Matthews, 1999, 2001a,b; Bozza et al., 2002). However, the dynamics of the transduction current is concentration-dependent and develops a faster transient component at higher concentrations (Menini et al., 1995; Reisert and Matthews, 1999). Driven by the transduction current, ORN firing rates also become more transient and the overall number of elicited spikes decreases for stronger stimuli, eventually reducing to only a few spikes at the onset of a high odor concentration (Getchell and Shepherd, 1978a,b; Duchamp-Viret et al., 1999, 2003; Reisert and Matthews, 1999, 2001a,b; Figure 3A). Current injection in ORNs induces much less complex firing dynamics (Ma et al., 1999) demonstrating a key role for the transduction current in determining ORN response dynamics.

Recording from isolated ORNs allows for the tight control of the stimulus and direct quantification of their electrical properties but shortcuts several steps that are important *in vivo*, such as respiration and the absorption of odor molecules in the mucus (Reisert and Matthews, 1999). For example, testing an odor response requires delivering odors in solution rather than as a volatile airborne stimulus. Electrode measurements from ORNs *in vivo* are possible (Duchamp-Viret et al., 1999), but the approach is not widely used due to the technical challenges posed by the physical organization of the ORNs and the location and structure of the olfactory mucosa (Duchamp-Viret and Chaput, 2018). Calcium imaging offers an alternative approach for quantifying ORN responses *in vivo*. ORNs can be anatomically labeled with organic dyes (Friedrich and Korsching, 1997; Ma and Shepherd, 2000; Wachowiak and Cohen, 2001; Fried et al., 2002; Wachowiak et al., 2002; Korsching, 2005), and genetic targeting strategies have used the olfactory marker protein (OMP) promoter (Farbman and Margolis, 1980; Danciger et al., 1989) to generate transgenic mice expressing different reporters of neural activity in ORNs (Bozza et al., 2004; McGann et al., 2005; Albeanu et al., 2018; Dewan et al., 2018; Platisa et al., 2020).

Functional imaging from single cells in the olfactory epithelium *in vivo* has reported monotonic relationships between calcium transients and odor concentration for many odor-receptor combinations (Inagaki et al., 2020, 2021; Xu et al., 2020; Zak et al., 2020). This is in agreement with the role of calcium in the initial amplification step in the signaling cascade (Leinders-Zufall et al., 1997, 1998). However, these measurements of somatic calcium dynamics did not show the transient activity measured in electrophysiological experiments (Reisert and Matthews, 1999, 2001a,b) and thus may not be an appropriate reporter of ORN firing rates.

How do peripheral firing dynamics translate to synaptic inputs to the bulb? Functional imaging has been widely used to quantify the neural activity measured from ORN axon terminals innervating the glomerular layer. ORN glomerular imaging trades single-cell resolution for a gain of information at the population level measured across the ~200 glomeruli that are located on the dorsal surface of the OB (Vincis et al., 2012). Measurements from the ORN glomerular projections reflect some combination of ORN firing activity as well as modulation by the OB network *via* presynaptic GABA and dopamine receptors expressed on the ORN axon terminals (Nickell et al., 1991, 1994; Wachowiak and Cohen, 1998, 1999; Koster et al., 1999; Berkowicz and Trombley, 2000; Ennis et al., 2001; McGann, 2013). In principle, pooling the response of the multiple ORNs within a single glomerulus (Cleland and Linster, 1999; Cleland et al., 2011; Cleland and Borthakur, 2020), as well presynaptic modulation at the axon terminals (Cleland and Linster, 2012; McGann, 2013) should broaden the dynamic range of the glomerular ORN response. However, a direct comparison between calcium transients measured from ORN somata and from their terminals in the OB revealed no major differences (Zak et al., 2020). Importantly, with one exception (Lecoq et al., 2009), all these studies reported primarily monotonic relationships between presynaptic activity and odor concentration and tonic responses dynamics, in contrast with the concentration dependency of ORN somatic firing patterns (Figures 3A,B). These differences could appear to be a minor discrepancy, but understanding the response dynamics of ORNs to changes in stimulus intensity is critical for understanding their adaptive features.

### Response to Dynamic Stimuli: What Goes Through the ORNs?

Natural odor stimuli are carried by air, often in turbulent regimes that break diffusive odor signals into filaments of different sizes. This has the effect of generating an intermittent and stochastic concentration profile (Celani et al., 2014; Connor et al., 2018). Moreover, in breathing animals, respiration modulates the sampling of an odor stimulus by controlling the times and durations of the odor plumes that reach the ORNs. Consequently, nearly no odor stimulus is sensed in complete isolation, and it is, therefore, crucial to understand how ORN responses are affected by stimulus history.

Paired-pulse experiments have been used to mimic the arrival of consecutive plumes or the intermittency imposed by respiration on odor perception. Measurements *in vitro* show that ORNs exhibit a form of adaptation that attenuates their response up to inter-stimulus intervals of 6–10 s (Kurahashi and Menini, 1997; Leinders-Zufall et al., 1998; Ma et al., 1999). This is a surprisingly long timescale and suggests that in freely breathing animals ORN responses should change drastically across breathing cycles (Figures 3C,D). As one would expect, this effect depends on the odor concentration and the stimulation or respiratory rate. ORNs reliably fire spikes *in vitro* at every odor pulse of a moderate concentration when delivered at 2 Hz intervals, but spiking is less reliable and firing responses attenuate and eventually disappear at higher stimulation rates (5 Hz) or at higher odor concentrations (Ghatpande and Reisert, 2011). Although an exact quantification of the ORN integration time has not been attempted in vertebrates, these experiments show that it likely falls in the range of the respiratory period (200–500 ms). Whether this is a limitation of the olfactory periphery in precisely encoding odor stimuli or serves a functional role in filtering sensory inputs sent to the brain remains unclear. The *in vitro* data suggest that for a fixed stimulation frequency, only the ORNs that are mildly activated by the odor would contribute to encoding the full stimulus sequence, while more sensitive ones would signal only the onset of the sequence and remain otherwise silent (Figure 3D, *compare ORN*1 and *ORN2*).

Stimulus-driven changes in response do not only originate at the periphery. *In vitro* and *in vivo* studies have shown that repeated electrical stimulation of the olfactory nerve (which shortcuts peripheral adaptation) affects the amount of glutamate released onto post-synaptic neurons (Aroniadou-Anderjaska et al., 2000; Ennis et al., 2001; Murphy and Isaacson, 2003). Consistently, calcium imaging from ORN terminals *in vitro* and *in vivo* reported adaptation to paired-pulse electrical stimulation of the olfactory nerve that recovered with interstimulus intervals longer than 1 s (McGann et al., 2005; Wachowiak et al., 2005; Pírez and Wachowiak, 2008). This adaptation appears to be due to depression at the level of the ORN terminals as it similarly affects simultaneously recorded post-synaptic neurons (Murphy et al., 2004). Application of a GABA antagonist reduced the effect of the paired-pulse depression and increased the rate of recovery to paired-pulse stimulation *in vitro* (Aroniadou-Anderjaska et al., 2000; Ennis et al., 2001; Wachowiak et al., 2005), suggesting a key role for inhibition in the temporal filtering of olfactory information. Similarly, studies in insects have shown that depression at the synapses between ORNs and PNs (the invertebrate homolog of MTCs) act on multiple time scales to filter incoming signals (Kazama and Wilson, 2008; Martelli and Fiala, 2019), while the dynamics of lateral inhibition controls the width of these filtering steps (Nagel et al., 2015). The dynamic interplay between feedforward depression and lateral inhibition remains to be investigated in the mammalian brain *in vivo*, especially in the context of adaptation in breathing animals.

The combination of peripheral and central mechanisms that attenuate the response to consecutive odor pulses should lead to a major change in the OB activation across consecutive respiratory cycles. However, *in vivo* imaging from the ORN terminals in awake and anesthetized rats and mice reported attenuation only for high respiratory frequencies (>4 Hz) and for specific odor receptor combinations (Verhagen et al., 2007; Carey et al., 2009; Carey and Wachowiak, 2011). Inter-stimulus intervals of 330–1,000 ms, which corresponds to respiratory frequencies of 1–3 Hz, have been reported to induce substantial depression *in vitro* (Kurahashi and Menini, 1997; Leinders-Zufall et al., 1998; Zufall and Leinders-Zufall, 2000; Wachowiak et al., 2005), but similar rates only evoke minor effects in the activity measured from the glomeruli *in vivo* (Verhagen et al., 2007; Carey and Wachowiak, 2011). It is unclear whether these inconsistencies are due to different experimental conditions or due to odor-driven and state-dependent presynaptic processing in the bulb. More specific approaches are needed to understand the relationship between the peripheral processes and the presynaptic activity in the bulb. One key open question is whether the OR expressed plays a significant role in determining the ORN response dynamics. If this is not the case, then one could conclude that differences in the activity of different glomeruli depend on lateral connectivity. Most of the earliest studies have been performed from randomly selected cells, but the development of transgenic animals with genetically labeled ORN types allows for a comparison of the same receptor types across preparations (Bozza et al., 2002; Grosmaitre et al., 2006; Ghatpande and Reisert, 2011). This approach could be further exploited to investigate receptor-specific response properties and would clarify the contribution of intrinsic cellular mechanisms that originate at the periphery and presynaptic modulation which is mediated by lateral inputs in the OB.

### Background Segregation and Contrast Detection in ORN Populations

A classical approach to study adaptive properties of sensory neurons is to compare the response to a stimulus presented in isolation and on a background. Experiments *in vitro* showed that adaptation to an odor background lowers the ORN transduction current, as well as the firing rate response to an odor (Reisert and Matthews, 1999, 2000). In adapted conditions, response saturation is reached at lower peak firing rates and at similar concentrations than in the absence of a background, causing a compression of the ORN dynamic range. Similar results were reported in *Drosophila* ORNs (Martelli et al., 2013; Brandão et al., 2021).

The functional consequences of a decreased dynamic range remain unclear. Such compression does not support contrast invariant responses, and therefore single ORNs likely do not signal stimulus contrast. However, the data indicate that in the presence of a background, ORN firing is more parsimonious, consisting of transient responses of a smaller number of spikes (Figure 3A). Therefore, at the population level, this could still constitute a strategy to efficiently signal changes in concentration and segregate relevant stimuli from the background. Indeed, odors are detected by a large array of ORNs, each expressing an odorant receptor with a different affinity to odor molecules. Therefore, the olfactory system is, by constitution, endowed with a large array of sensors tuned to different intensities of the same stimulus (i.e., different concentrations of a monomolecular odorant; Cleland et al., 2011; Zak et al., 2020). For example, when the background is too high an ORN will go silent, rather than shifting its dynamic range, which is energetically convenient given that the system can rely on the activation of other ORNs of lower sensitivity. Thus, **adjusting the dynamic range of the response of single sensory neurons may not be the primary function of adaptation in olfaction**.

Such hypotheses should be tested at the level of the OB by quantification of the population response to stimuli presented in isolation or on a background. However, the stimulus protocol can hardly be the same *in vivo* and *in vitro*, as respiration adds a level of complexity to the encoding of odor information. Respiratory frequency determines the degree by which adaptation affects the response to a chemically *different* odor superimposed on a background (Verhagen et al., 2007). At low inhalation rates, glomeruli that are sensitive to both background and test odors responded strongly to their superposition, but higher respiratory rates caused significant adaptation to the background and a highly attenuated response to the test odor. Unfortunately, the interpretation of these results cannot be solely based on adaptation, as mixture interaction at the level of olfactory receptor activation and at the OB local network could have effects on the observed dynamics (Inagaki et al., 2020; Xu et al., 2020; Zak et al., 2020). However, the data suggest so far that at low respiratory frequencies, ORNs have enough time to recover from adaptation during the exhalation phase. This would mean that tuning respiration could be used as a tool to adapt and dis-adapt the sensory neurons, facilitating background segregation in one direction and enhancing mixture integration in the other. Stimulus-specific adaptation should be further investigated using background stimuli of the same chemical identity as the test one.

### The Controversial Function of Molecular Mechanisms Involved in ORN Adaptation

The molecular bases of olfactory transduction have been investigated extensively over many decades. In short, calcium has been identified as a key player in suppressing ORN responses in paired-pulse experiments (Zufall et al., 1991; Kurahashi and Menini, 1997). The calcium-calmodulin (Ca^2+^-CaM) complex had been originally proposed as the main modulator of cAMP affinity to the CNG channels (Chen and Yau, 1994; Bradley et al., 2001). However, a subsequent study showed that the Ca^2+^-CaM feedback does not regulate cAMP sensitivity of the channel, but rather controls response termination (Song et al., 2008). Similarly, a genetic approach has revealed a minor role for the Ca^2+^-CaM feedback on ACIII (Reisert and Zhao, 2011), a molecular pathway that had been initially proposed as a mechanism to adapt sensitivity in the presence of prolonged stimulation (Leinders-Zufall et al., 1999). Mechanisms involved in the hydrolyzation of cAMP and in the removal of ciliary calcium have been proposed as important regulators of ORN response termination that requires the closing of CNG channels and calcium dependent Cl^−^ channels (Reisert and Zhao, 2011).

Whether it is possible to mechanistically separate response termination from an adaptation of sensitivity remains unclear. These two phenomena remain coupled if their dynamics are not taken experimentally apart. Paired-pulse experiments, for example, do not allow a net separation of the two mechanisms as the response to following stimuli could be lowered either by a slow response termination or by a change in sensitivity. However, this is crucial in the context of breathing animals, and it makes sense that the olfactory system has implemented mechanisms to control the speed of response onset and termination (rather than to adjust sensitivity), as this is fundamental to the perception of stimuli wrapped in the inhalation phases. Failure to keep up with respiration can lead to unreliable responses. Whether these response mechanisms are adaptive in the sense that their properties adjust to the respiratory cycle or are just limited by respiratory frequency remains unclear. An analysis of the temporal aspects of ORN firing rate responses with more complex stimuli is needed to further understand the peripheral processing of odor stimuli and the specific role of identified molecular pathways.

## Spatiotemporal Features of Adaptation in Populations of MTCs

### Reformatting Information About Stimulus Concentration in Populations of MTCs

Experiments across a variety of different model organisms, preparation types, and experimental techniques reported a complex and heterogeneous relationship between MTC activity and odor concentration. Individual cells exhibit excitatory or inhibitory responses and cell-specific temporal dynamics to different concentrations of the same odor. Electrophysiological studies have shown that there is no obvious rule for the encoding of stimulus concentration in the firing rate of single MTCs (Sirotin et al., 2015). Some MTCs show monotonic (increasing or decreasing) responses to changes in stimulus concentration, some MTCs show nonmonotonic responses, and others have concentration invariant responses (Mathews, 1972; Kauer, 1974; Kauer and Shepherd, 1977; Meredith and Moulton, 1978; Mair, 1982; Meredith, 1986; Reinken and Schmidt, 1986; Chaput and Lankheet, 1987; Hamilton and Kauer, 1989; Motokizawa, 1996; Chalansonnet and Chaput, 1998; Niessing and Friedrich, 2010; Banerjee et al., 2015; Yamada et al., 2017). Similar diversity is observed in 2-photon imaging experiments that quantified activity from the MTC dendritic arborizations in the glomerular layer (Economo et al., 2016; Moran et al., 2019; Storace et al., 2019). This range of concentration dependence across MTCs is strikingly wider than what has been observed in the ORN input to the glomerular layer, which is mostly monotonic with odor concentration (Figure 3B). This suggests a major function of the OB circuit in shaping the encoding of odor concentration.

**Figure 4:**
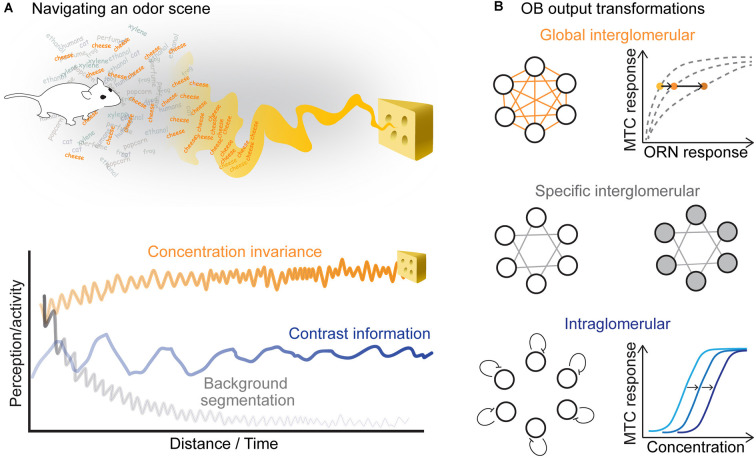
**(A)** In order to localize an odor source, the animal should keep a concentration invariant representation of the odor stimulus, while tracking its changing concentration and segmenting the source-specific smell from other background odors. **(B)** Three kinds of transformations that occur in the olfactory bulb could support these computations. (*Top*) Global interglomerular inhibition performs a divisive normalization of the OB output. Increasing odor concentration increases feedforward excitation and lateral inhibition leading to a concentration invariant representation in MTCs. (*Middle*) Specific interglomerular inhibitory inputs may support background segmentation. (*Bottom*) Intraglomerular inhibition could shift the sensitivity of MTCs, which would allow them to retain contrast information in different odor backgrounds.

Maintaining both a concentration-invariant representation of an odor as well as concentration-specific information are both critical for robust and flexible behavior (Figure 4A). Can these aspects of an odor stimulus be decoded from the population activity of the OB rather than from single MTCs? Clearly, information about absolute concentration is not discarded in the OB since animals can discriminate different concentrations of the same odor (Jordan et al., 2018a) and use concentration information for tracking an odor to its source (Catania, 2013; Findley et al., 2021). Information about concentration is retained in the combinatorial activity of the glomeruli (Storace and Cohen, 2017; Storace et al., 2019). Higher odor concentrations not only recruit ORNs with lower sensitivity (Wachowiak and Cohen, 2001; Bozza et al., 2004) but also drive more lateral inputs in the OB (Banerjee et al., 2015; Storace et al., 2019). Thus, concentration changes will activate both excitatory and inhibitory synapses, which will affect the overall MTC population activity. In zebrafish, MTC population responses have been shown to be robust within a certain range of odor concentrations (Niessing and Friedrich, 2010). However, studies in mice did not confirm these observations and rather show that different odor concentrations elicit a continuum of distinguishable representations at the population level (Bathellier et al., 2008). Therefore, it seems too simplistic to try to assign a single function to the OB circuit.

There are at least three types of lateral inputs with possibly different functions in the OB (Figure 4B). First, lateral inhibitory neurons that broadly innervate the OB rescale the excitatory feedforward responses of individual glomeruli by the overall activation of the bulb (Cleland and Sethupathy, 2006; Banerjee et al., 2015). Similar to the insect antennal lobe network, this kind of connectivity could support a canonical computation, named divisive normalization (Olsen et al., 2010; Carandini and Heeger, 2011). Theoretical considerations suggest that divisive normalization may lead to concentration invariant representations in the OB output (Figure 4B, *top*). Dual-color voltage and calcium imaging have shown that odor representations become indeed more concentration-invariant across this synaptic step (Storace and Cohen, 2017; Storace et al., 2019). However, in addition to broad inhibitory inputs, several examples of selective inhibitory inputs have been reported, which most likely play a key role in shaping odor-specific OB output activity patterns (Fantana et al., 2008; Economo et al., 2016). As proposed in a model of the OB (Koulakov and Rinberg, 2011), this kind of inhibitory feedback could lead to transient output responses and, therefore, enhance segregation of background from foreground stimuli (Figure 4B, *middle*). Finally, the OB output is modulated by high-sensitivity periglomerular interneurons that can suppress MTC responses to weak ORN input within a single glomerulus, shifting their sensitivity to higher concentrations (Gire and Schoppa, 2009; Cleland and Linster, 2012). This sensitivity shift could, in principle, support contrast invariant responses of MTCs (Figure 4B, *bottom*). These three computations probably run in parallel, as none of them seem to be sufficient to explain the response of all MTCs.

We conclude that, even though the architecture of the OB circuit can support both concentration invariance, background segmentation, and contrast encoding (Figure 4), it remains unclear whether these three computations lead to distinct parallel readouts at the level of the OB or rather require further processing downstream of the OB. Better insight into the function of the OB circuit should be obtained by looking at the adaptive changes in the function of these circuit motifs and how they shape MTC responses to sustained and repeated stimulation.

### Temporal Properties That Lead to Integration and Differentiation of Odor Stimuli

How do MTCs respond to a sustained odor stimulus? Because subsets of MTCs are strongly coupled to inhalation, population activity on short timescales is driven by the respiration frequency, and population dynamics elicited by a continuous odor stimulus evolves on cyclic patterns (Bathellier et al., 2008). Single MTCs show a large diversity of responses across these breathing cycles. Relatively sustained spiking activity has been observed in rats when using an artificial sniffing paradigm at rates between 2–5 Hz (Sobel and Tank, 1993), and in freely breathing rats in response to 40–50 s long odor stimuli (Wilson, 1998; Cang and Isaacson, 2003; Kadohisa and Wilson, 2006). However, other studies reported marked attenuation of MTC firing rates in the presence of continuous or repetitive odor stimulation (Potter and Chorover, 1976) and similarly in freely breathing mice across respiratory cycles (Meredith and Moulton, 1978; Døving, 1987; Sobel and Tank, 1993; Wilson, 2000; Margrie et al., 2001; Sirotin et al., 2015; Bolding and Franks, 2018; Ogg et al., 2018; Moran et al., 2019; Parabucki et al., 2019).

Can the large diversity of temporal integration properties in MTCs be quantified? Similarly to results from insect olfactory projection neurons (Geffen et al., 2009; Martelli and Fiala, 2019), convolution of the stimulus with a linear filter is sufficient to predict the response of single MTCs to odor pulses of different lengths and temporal dynamics (Gupta et al., 2015). Each MTC-odor pair can be fitted by a linear filter of a specific shape: some with a positive polarity indicating excitatory responses, and some with a negative polarity indicating inhibitory responses. In most cases, these linear filters are biphasic (i.e., with a positive lobe followed by a smaller negative one, or vice versa), which suggests that these neurons calculate a derivative of the incoming stimuli. In other words, at least a subset of MTCs respond to changes in concentration and adapt their firing to a sustained stimulus.

Although the shape of the linear filters is variable across cells, in most cases they are about 0.5–1 s wide, indicating that filtering and integration of the stimulus occur on timescales longer than a single respiratory cycle. This is consistent with the observation that population dynamics evolve on timescales longer than the respiratory frequency (Bathellier et al., 2008). On the contrary, MTC linear filters extracted in response to current injection when all synaptic inputs were blocked, have much shorter integration times on the order of 20 ms (Padmanabhan and Urban, 2010), which argues for a major role of synaptic inputs in determining MTC dynamics on longer timescales. The linear filters however do not predict the response to different concentrations of the same stimulus, confirming again the non-linearity of MTC responses to odor intensity and suggesting that scaling odor concentration does not simply scale feedforward signals. Importantly, MTC responses in breathing animals can be predicted by the convolution of the cell-odor specific filter with the respiratory flow (Gupta et al., 2015), and therefore the actual odor reaching the receptor neurons can be assumed to be a simple product of odor stimulus and respiration. This model predicts that if the breathing period is shorter than the filter width, then the response to consecutive inhalations will fuse, resulting in a reduced locking of the response to respiration. Different degrees of respiration locking have been observed across individual MTCs (Patterson et al., 2013; Eiting and Wachowiak, 2020), but whether this linear model captures such a broad spectrum of properties has not been tested. Specifically, mitral cells exhibit longer response durations than tufted cells (Short and Wachowiak, 2019), and tufted cells are more locked to respiration (Nagayama et al., 2004; Igarashi et al., 2012; Díaz-Quesada et al., 2018; Eiting and Wachowiak, 2020). One interesting possibility is that the degree of respiration locking depends on the integration timescale (i.e., the width of the linear filter) of different cells. A shorter integration time would lead to stronger respiratory coupling, but a lower capability to compare stimuli across respiratory cycles. Whether these different dynamics could reflect intrinsic properties of mitral and tufted cells or different synaptic inputs in these cell types remain an open question.

Further analysis is required to identify cell-intrinsic and network mechanisms that determine the different temporal properties of mitral and tufted cells. But do these properties imply that different information about the stimulus is encoded in different MTCs?

### Different Subsets of MTCs Encode Contrast, Concentration, and Intensity Invariant Information

Delivering an isolated odor pulse involves presenting a new chemical at some concentration. Is the response of a neuron determined by the specific odor, its absolute intensity, or the relative change in concentration compared to the background? Very few studies have quantified how MTCs respond to changes in odor stimuli starting from an adapted state. One study reported that MTCs show minimal adaptation to a background odor and respond to the addition of a second odor as the sum of the response to the two odors presented individually (Kadohisa and Wilson, 2006). However, this seems to be a special case. More recent investigations clarified that generally, background odor adaptation significantly affects MTC responses in a cell- and odor-dependent manner (Vinograd et al., 2017; Parabucki et al., 2019). Three types of MTCs have been identified based on their functional response to step increases in odor concentration. Type I encodes absolute concentration, type II is concentration invariant, and type III responds to relative changes in concentration (Parabucki et al., 2019). These, however, are not genetically distinct cell types with assigned functions, as the same MTC can be concentration invariant or encode concentration changes depending on the specific stimulus delivered. These observations are therefore consistent with the diversity of dynamics described in the previous paragraph, with a range of capabilities to differentiate and integrate incoming stimuli in a cell- and odor-specific manner.

One possibility is that the OB network is designed to encode different features of a smell in different subsets of MTCs: stimulus intensity in type I responses, stimulus identity in type II responses, and stimulus contrast in type III responses. But further experiments are needed to understand whether these categories are robust to larger concentrations ranges, respiration rates, and odor identity. Since these functional classes of MTC responses do not seem to define cell types, they are most likely determined by lateral connectivity and the specific activation within their local network. One exciting possibility is that the majority of lateral inputs on a MTC differs in different contexts (Figure 4B). This hypothesis further raises the question of whether these MTC subtypes belong to the same glomerulus or rather sibling MTCs from the same glomerulus are wired to inhibitory neurons of different classes.

### Temporal Decorrelation and Categorization of Odor Representations

Following the diverse dynamics of single neurons, the odor representation in the population of MTCs evolves spatially over breathing cycles. Several studies have shown that the correlation between the current population response and the initial response decays over breathing cycles (Cleland and Linster, 2012; Patterson et al., 2013; Friedrich and Wiechert, 2014; Díaz-Quesada et al., 2018; Eiting and Wachowiak, 2020). This indicates an overall reorganization of the OB activity that is not a simple linear attenuation. But where does this transformation lead to? One possibility is that odor representations evolve based on a categorization process, somehow intrinsic to the OB network connectivity and driven by the coactivation of certain glomerular patterns. Morphing experiments have been used to quantify the degree of categorization within the OB using a continuum of proportional mixtures of two odors. In Zebrafish it was shown that temporal decorrelation in population activity supports the classification of gradually changing stimuli into discrete output patterns (Niessing and Friedrich, 2010). However, a similar approach in rats reached opposite conclusions (Khan et al., 2008), suggesting that categorization might or might not occur depending on the specific concentration range or odorant mixtures used. Even so, it remains clear that this time-dependent and stimulus-induced population plasticity could help disentangle temporally intermingled odor stimuli as they occur in the wild (Figure 4A). One possibility is that the autocorrelation of distinct odor sources will drive adaptation in different odor-specific sets of coactivated glomeruli that will independently evolve in different directions in the response space, leading to the separate categorization of coherent components from non-coherent ones. This process would support background segregation of complex chemical mixtures.

### Active Sampling as a Mechanism to Tune Adaptation in The Olfactory Pathway

**Figure 5:**
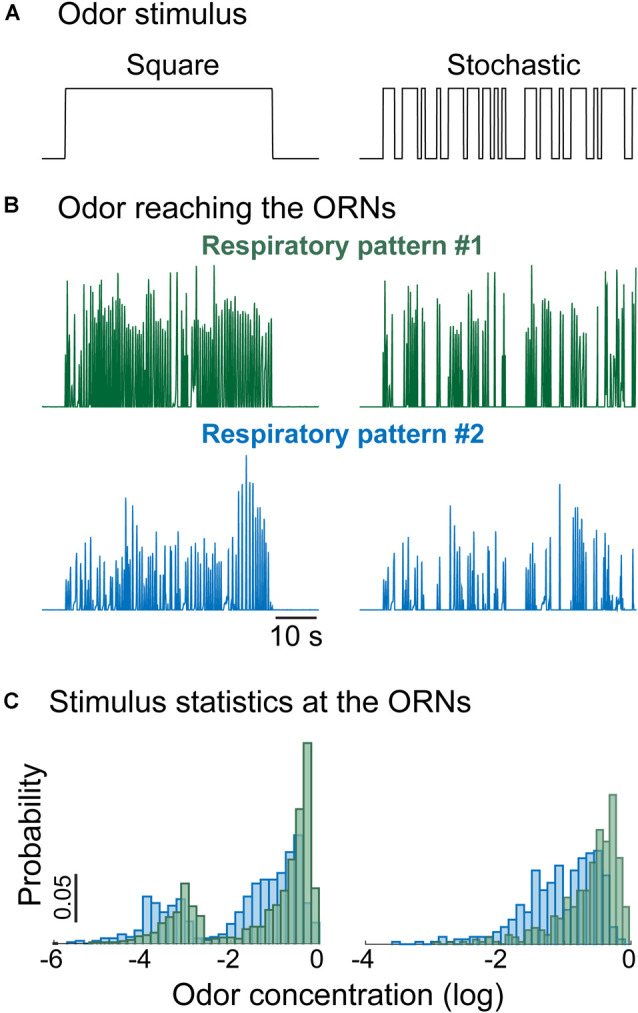
Respiratory rate shapes the statistics of the stimulus reaching the ORNs. **(A)** Example of odor stimulus reaching the animal: (*left*) constant concentration, (*right*) random flickering odor stimulus with the same fixed concentration. **(B)** Time-dependent odor concentration reaching the ORNs calculated as the product between the inhalation phase of the normalized respiratory trace and the odor stimulus. **(C)** Distribution of the odor concentration reaching the ORNs. Different stimulation protocols and respiratory patterns give rise to different distributions even though the delivered odor concentration was the same.

One intriguing aspect of olfactory coding in breathing vertebrates is the capability of animals to modulate respiration in a task-dependent manner to sample odor stimuli (Verhagen et al., 2007; Wesson et al., 2008; Koldaeva et al., 2019). Differences in respiration determine differences in the statistics of the perceived odor stimuli. The example in Figure 5 shows that the mean stimulus intensity, as well as the shape of the stimulus distribution, strongly depend on the respiration pattern [here we assumed that the odor concentration activating the ORNs is a simple product of odor stimulus and inhalation airflow (Gupta et al., 2015)]. The distribution of the inputs determines the degree of adaptation and stimulus-driven plasticity in sensory pathways, and therefore **modulation of sniffing changes both the input stimulus as well as the OB network state that processes incoming stimuli**.

As with ORNs, the respiratory rate also influences MTC activity (Carey and Wachowiak, 2011; Díaz-Quesada et al., 2018) with diverse effects across individual MTCs. Increased respiration rate can drive a decrease or an increase in firing rate, or in some cases induce a higher degree of locking to the inhalation phase. The timescales on which these changes occur have not been explicitly quantified, but it seems unlikely that the data can be explained with a single mechanism. Rather, a range of different mechanisms that act on multiple timescales up to several seconds seems to be activated depending on the respiration rate (Díaz-Quesada et al., 2018). These observations challenge the linear filter model of MTC responses, which would only fit dynamics up to 1 s, and suggest that they probably only capture steady-state properties of MTCs. While some of the stimulus-induced changes in MTCs may reflect ORN adaptation (Ogg et al., 2015), MTCs can exhibit attenuated responses even when the corresponding presynaptic input is sustained (Storace and Cohen, 2019). Moreover, the preferred phase of individual MTC responses shift with inhalation frequency (Díaz-Quesada et al., 2018), a property that has not been observed in ORNs and is thus unlikely to be inherited from the periphery.

In general, higher inhalation frequencies result in stronger changes in MTC activity, but other parameters such as inhalation duration or amplitude might play an important role in freely breathing animals (Courtiol et al., 2011a,b). Changes in sniffing can mimic the effect of increased concentration on MTC firing, even though at the behavioral level information about odor concentration remains intact (Jordan et al., 2018a). This implies the existence of a mechanism to decouple the effect of respiration from the odor stimulus and suggests an important role of respiratory-driven MTCs in odor information transmission. Animals tune their respiration in a task-specific manner (Jordan et al., 2018b), and therefore, respiration could be modulated to shape the statistics of the perceived odor stimulus without compromising its information content (Figure 5). It would be interesting to understand how the different features of respiration (i.e., inhalation frequency, duration, and amplitude) differently affect adaptation in olfactory processing. In principle, longer inhalation should drive more activity-dependent plasticity, while higher frequencies should engage mechanisms for response termination and, finally, inter-inhalation times should allow for recovery from adaptation. Respiration is a filter imposed on a stimulus already rich in dynamics, although the stimulus properties that drive a change in respiration remain unclear. This feedback modulation of the sampling strategy is likely accompanied by top-down processes that serve to modulate odor inhalation and odor processing in a task-specific manner (Reisert et al., 2020), and based on the saliency of the specific odor and previous experience (Wesson et al., 2008; Jordan et al., 2018b).

### Cell Intrinsic and Network Mechanisms Underlying MTC Adaptation

Adaptation of MTCs is to some degree due to adaptation inherited from presynaptic ORNs and further modulated by properties of feedforward synapses (such as depression). However, three other mechanisms might contribute to the adaptive responses of MTCs: (1) cell-intrinsic physiological mechanisms; (2) integration of lateral inputs; and (3) feedback modulation from other brain areas.

MTCs express many different receptors and channels which underlie diverse intrinsic biophysical and functional properties (Angelo and Margrie, 2011; Angelo et al., 2012). Injecting current into MTCs can evoke diverse responses from periodic bursts of firing (Chen and Shepherd, 1997; Desmaisons et al., 1999; Balu et al., 2004), to more sustained spiking activity that gradually declines (Fadool et al., 2011; Tucker et al., 2013; Burton and Urban, 2014). These properties are still visible when synaptic transmission is pharmacologically blocked suggesting that they reflect intrinsic biophysical properties of MTCs (Burton and Urban, 2014). MTCs express the Kv1.3 potassium channel (Fadool et al., 2000, 2004), which can be locked in an inactive state in response to repeated stimulation (Marom and Levitan, 1994) and could play a role in adaptation. However, Kv1.3 knockdown also changes the OB glomerular structure (Fadool et al., 2004), therefore the precise functional role in odor encoding is hard to pinpoint. It would be interesting to know whether differences between mitral and tufted cells can be attributed to different expression levels of this or other channels.

The dynamic evolution of OB odor representations is certainly strongly determined by the dynamics of specific types of lateral inputs. MTC adaptation was reduced by the application of the GABA-A antagonist bicuculine (Margrie et al., 2001), and similarly by the NMDA antagonists MK-801 *in vivo* (Chaudhury et al., 2010). Moreover, lateral inhibition acts with its own temporal dynamics. For example, synapses between GABAergic granule cells and mitral cells exhibit significant paired-pulse depression for inter-stimulus intervals up to 10 s (Dietz and Murthy, 2005). Understanding the dynamics of different inhibitory neurons and manipulating their connectivity in a cell-specific manner would shed light on their functional role in the context of olfactory adaptation.

Finally, the OB receives feedback projections from other brain areas (Macrides et al., 1981; Luskin and Price, 1983; Petzold et al., 2009; Rothermel et al., 2014; In’t Zandt et al., 2019; Padmanabhan et al., 2019; Schneider et al., 2020), some of which are related to state-dependent modulation (Linster and Cleland, 2016; McIntyre et al., 2017), for example in the context of feeding regulation (Pager et al., 1972; Wu et al., 2020), and therefore are not strictly stimulus-driven. Centrifugal feedback to the bulb can modulate the response to sustained or repeated odor presentation on long timescales, between minutes or days in the context of habituation (Wilson, 2009; Ogg et al., 2018), associative learning (Kiselycznyk et al., 2006), or context-specific behavior (Yamada et al., 2017). Most of these studies, however, focused either on response attenuation or pattern separation in the context of odor discrimination and stimulus salience. An exciting possibility is that behavioral state or behavioral outcome can alter temporal processing of odor stimuli in the OB *via* these feedback pathways, similarly to behaviorally dependent processing that has been described in the visual system (Maimon, 2011). However, the specific role(s) of centrifugal mechanisms in adaptive coding remains to be investigated.

## Conclusions and Outlook

This review was motivated by the need to define the current understanding of the role of adaptation in olfactory coding and to identify the directions in which further research should be aimed to link cellular and circuit mechanisms to their behavioral function. In other sensory systems, peripheral adaptation mediates a shift of the sensory response to match stimulus statistics (Figure 1A). However, only a handful of studies have experimentally approached olfactory adaptation asking whether it fulfills a similar role. This is likely due to the combinatorial and temporal complexity of the olfactory system. Combinatorial complexity is associated with the large repertoire of odorant receptors, which participate in odor coding when their sensitivity matches the stimulus concentration. Temporal complexity is related to the nature of the stimuli (volatile molecules transported by air flow), as well as to the filtering function applied by respiration in breathing animals. These two special aspects of the olfactory system suggest that we should think about adaptation differently than in other sensory modalities. What can we conclude from these observations? First, the computational role of peripheral ORN adaptation does not seem to be a shift in sensitivity. From electrophysiological studies, strong stimulus intensity, as well as sustained stimulation, reduces ORN responsiveness, either through saturation or adaptation. Although ORN adaptation has been quantified in a relatively small number of experimental paradigms, the data mostly support a model in which ORN adaptation reduces coding capacity by compressing the dynamic range of the response (Figure 1B). In this context, it remains unclear to which degree ORN firing rates encode stimulus intensity or contrast and further experiments are necessary to answer this question. Moreover, mechanisms that had been proposed to mediate a change in response sensitivity have been subsequently associated with response termination, a crucial step to control the temporal precision of odor perception, specifically in breathing animals. The field certainly calls for a better characterization of ORN firing dynamics by means of more complex stimuli that would mimic natural statistics and the modulations imposed by respiration. The possibility to genetically target specific ORNs should further unveil the degree of diversity of responses across the repertoire of receptors.

Our second observation is a degree of inconsistency between ORN response dynamics reported in electrophysiological studies and those quantified *in vivo* from the corresponding calcium transients in imaging experiments in the bulb (Figure 3). If we were to extrapolate from firing rate properties of single ORNs, we would predict a major rearrangement of ORN population activity depending on the stimulus. For example, the response of the most sensitive glomeruli should be extremely transient because at saturating concentrations ORNs only fire a few spikes and then go silent. Similarly, adaptation to a background should lead to no response from the most sensitive glomeruli. On the contrary, functional calcium imaging studies in the OB reported more robust and less adaptive ORN responses than expected. Although this reflects in some degree methodological differences, we believe that a major role is played by multimodal regulation of presynaptic calcium signaling. Studies in insects have attributed a major role in odor coding to presynaptic processing. In vertebrates, ORN glomerular activity is often considered the *input* into the olfactory system, however, it should be investigated as the *output* of the first processing step in olfaction. Specifically, it would be important to clarify how molecular mechanisms for ORN response adaptation and termination identified *in vitro* affect calcium dynamics in the bulb *in vivo*, especially in the context of combinatorial coding and breathing modulation. Additionally, the dynamics and adaptive properties of lateral inputs on ORN presynaptic terminals deserve further investigation.

Our third observation is that coding principles of single MTC responses hardly generalize to other MTCs. This population of cells is diverse not only intrinsically (mitral and tufted cells are genetically different) but also functionally. The function of single MTCs depends on the specific stimulus used and the animal’s state (e.g., respiration) and therefore can be only interpreted with respect to the whole population. In this context, asking whether single MTCs adapt to sustained stimuli by shifting or by compressing their dynamic range might not be the right question. One possibility is that MTCs can be flexibly assigned to different subpopulations with distinct functions: reporting breathing rate, encoding odor identity, or changes in concentration. These functional differences should be associated with different temporal properties and possibly regulated by the activation of lateral inputs in a stimulus-specific manner. This points to the need of analyzing the coding properties of single MTCs while selectively perturbing synaptic inputs.

While the different coding functions of single MTCs might reflect the way in which they integrate synaptic inputs on short timescales (<1 s), there is plenty of evidence that MTC responses evolve over longer times (1–10 s) with a consequent rearrangement of the combinatorial representation. The timescales on which these changes occur depend on breathing rate, indicating that they are indeed stimulus-driven and controllable by the animal (Figure 5). This poses two important questions: **what is changing in terms of information content (about the current stimulus) and what is changing in terms of coding capacity (of future stimuli) at the population level?** One possibility is that these adaptive changes mediate task-specific categorization of the odor representation. For example, in morphing experiments, adaptive changes can support the formation of mixture categories, in odor-background segregation tasks the identification of the stimulus of relevance, and in learning experiments the separation of rewarded from not-rewarded stimuli. These situations will differentially enroll stimulus-driven feedforward mechanisms (synaptic depression and lateral local inputs in the OB) and state-driven top-down regulation (from cortical areas to the OB). While tracking an odor cue to the source, modulation of sniffing could be used to better separate the target odor from a background by tuning the degree of adaptation in the OB circuit and following the increasing gradient as encoded in MTC subpopulations. Understanding the physiological bases and context-dependent role(s) of these adaptive mechanisms remains the major goal in the field.

## Author Contributions

CM and DS both contributed to writing of the article. All authors contributed to the article and approved the submitted version.

## Conflict of Interest

The authors declare that the research was conducted in the absence of any commercial or financial relationships that could be construed as a potential conflict of interest.

## Publisher’s Note

All claims expressed in this article are solely those of the authors and do not necessarily represent those of their affiliated organizations, or those of the publisher, the editors and the reviewers. Any product that may be evaluated in this article, or claim that may be made by its manufacturer, is not guaranteed or endorsed by the publisher.
